# Antimicrobial photodynamic therapy with 5-aminolevulinic acid plus antibiotics: a promising treatment for tibial osteomyelitis caused by drug-resistant bacteria

**DOI:** 10.3389/fphar.2025.1566744

**Published:** 2025-03-10

**Authors:** Ju Zuo, Feiyan Kong, Xiyu Wang, Tianyu Wang, Jianxi Zhao, Zhanjuan Zhao

**Affiliations:** ^1^ Department of Radiology, Affiliated Hospital of Hebei University, Baoding, China; ^2^ School of Clinical Medicine, Hebei University, Baoding, China; ^3^ School of Basic Medicine, Hebei University, Baoding, China

**Keywords:** animal model of osteomyelitis, osteomyelitis, drug-resistant bacteria, photodynamic antimicrobial chemotherapy, microimaging indicators, micro-CT

## Abstract

Osteomyelitis is a severely destructive bone disease caused by microbial infections, and currently, no available treatment effectively controls the infection. 5-Aminolevulinic acid is a second-generation endogenous photosensitizer. This study investigated the efficacy of 5-aminolevulinic acid-mediated photodynamic therapy (ALA-PDT) in combination with antibiotics in the treatment of tibial osteomyelitis in rabbits. The results illustrated that ALA-PDT alone and in combination of antibiotics displayed significant efficacy in treating osteomyelitis. Animals in the photodynamic antimicrobial chemotherapy (PACT) + antibiotics group exhibited a higher survival rate, an improved overall mental status, a lower localized infection rate, and reduced Tang Hui and Norden scores (P < 0.05), indicating less severe bone destruction. Histologically, more strips of lamellar new bone formation and more pronounced periosteal hyperplasia were noted in the PACT + antibiotics group. Micro-computed tomography illustrated that the structural integrity of cortical bone and cancellous bone structure had better continuity and clearer display in the PACT + antibiotics group than in the other groups, and the periosteal reaction in the modeling area was the most obvious. Bone parameter analysis indicated that trabecular thickness, bone volume, and trabeculae volume were significantly higher in the PACT + antibiotics group than in the model and antibiotics groups (P < 0.05). Additionally, trabecular separation was significantly lower in the PACT + antibiotic group than in the other groups (P < 0.05). These findings suggest that the combination of ALA-PDT and antibiotics has a sensitizing therapeutic effect, offering a promising strategy for the clinical treatment of osteomyelitis.

## Introduction

Osteomyelitis is an infectious inflammatory disease characterized by bone destruction, and it is caused by pathogenic bacterial infections (Lew and Waldvogel, 2004). The infection primarily affects bone marrow, but it can also involve the bone cortex, periosteum, surrounding soft tissues, and adjacent joints. Inflammation can occur following surgery, trauma, infection, or skin degradation attributable to vascular insufficiency or peripheral neuropathy. Osteomyelitis is a persistent and challenging condition to manage, often leading to progressive bone destruction and osteonecrosis. In severe cases, amputation can be required. Effective control of both local and systemic infections is a fundamental principle in treating osteomyelitis in adults. Despite advances in research, clinical progress in the prevention and treatment of skeletal infections has been limited in recent decades, with the incidence of osteomyelitis continuing to rise (Muthukrishnan et al., 2019; Masters et al., 2022).

For instance, one study reported a 15.3% increase in the incidence of osteomyelitis in Germany between 2009 and 2019 (Walter et al., 2022). Osteomyelitis carries a significant physical and psychologic burden on patients and their families and imposes a substantial burden on healthcare systems. *Staphylococcus aureus* remains the predominant pathogen responsible for osteomyelitis (Urish and Cassat, 2020). However, widespread misuse of antibiotics has contributed to a dramatic increase in antimicrobial resistance globally (Ventola, 2015). In some regions, more than 50% of cases of osteomyelitis are caused by *Methicillin-resistant Staphylococcus aureus* (MRSA) (Turner et al., 2019; Yi et al., 2021). It is estimated that drug-resistant MRSA could cause nearly 10 million deaths annually by 2050 if no new anti-infective treatments are developed (Sutradhar et al., 2021).

In comparison with infections caused by other pathogens, osteomyelitis attributable to MRSA often has a more aggressive and complicated course. It is typically associated with higher inflammatory markers, more frequent febrile episodes, prolonged hospitalization, and an increased need for reoperations (Hawkshead et al., 2009). Additionally, MRSA-induced osteomyelitis is linked to a range of complications, including multi-organ failure, deep vein thrombosis, sepsis, pulmonary embolism, subperiosteal abscesses, and fractures (Belthur et al., 2012),which further complicate its management.

Osteomyelitis can be classified into acute, subacute, and chronic stages based on the disease’s duration (Jones et al., 2009; Obremskey et al., 2020). The pathophysiologic changes associated with osteomyelitis vary by stage, and treatment strategies differ accordingly (Waldvogel et al., 1970). Acute osteomyelitis typically develops rapidly and presents with clinical symptoms such as severe pain, swelling, elevated skin temperature, and localized tenderness. Early diagnosis is crucial for timely intervention. Acute osteomyelitis is usually managed with empirical antibiotic therapy and local decompression or drainage (Cierny et al., 2003). If left untreated or inadequately treated, it can progress to subacute osteomyelitis.

Subacute osteomyelitis often has an insidious onset and mild symptoms, and it lacks the characteristic clinical manifestations of acute osteomyelitis (St Jeor et al., 2020; Salik et al., 2021; Zairi et al., 2021). Although positive results are observed on imaging, its specificity is relatively low, which leads to the disease being easily misdiagnosed or missed in the clinic, thus delaying treatment. If this phase is overlooked and allowed to progress, chronic osteomyelitis can develop, often accompanied by biofilm formation, which is one of the hallmarks of chronic osteomyelitis. Once a biofilm forms, bacterial resistance to antibiotics increases significantly, and eradication of the infection generally requires surgical intervention (Cierny et al., 2003; Masters et al., 2019). Chronic osteomyelitis is a long-lasting condition, often persisting for months or even years because of severe bone destruction and various complications in later stages (Qian et al., 2020; Lari et al., 2024). This phase is particularly difficult to treat, and it carries a high risk of recurrence and no guarantee of complete resolution. Therefore, the key to effective osteomyelitis treatment lies in effectively arresting the progression of the disease early in its course. Early treatment of osteomyelitis focuses on infection control, but conventional antibiotic therapies are often insufficient. Furthermore, inappropriate antibiotic use can contribute to the emergence of drug-resistant bacterial strains (Kavanagh et al., 2018; Cortés-Penfield and Kulkarni, 2019).

Exploring novel therapeutic strategies that effectively manage infections while minimizing the risk of resistance development is crucial. Photodynamic antimicrobial chemotherapy (PACT) represents an innovative chemotherapeutic approach in which light irradiation triggers localized microbial inactivation. Unlike conventional bactericidal methods, the probability of bacterial resistance to PACT is significantly reduced (Sobotta et al., 2019; Maldonado-Carmona et al., 2020).

The three essential components of PACT are an excitation light source, a photosensitizer, and oxygen (St Denis et al., 2011), which together produce significant cytotoxicity. PACT is primarily performed using specific wavelengths of light to activate photosensitizers and generate reactive oxygen species (ROS). These ROS participate in cytotoxic reactions, oxidizing and destroying surrounding biomolecules, thereby killing pathogenic microorganisms (Kolarikova et al., 2023). The selection of a photosensitizer is a crucial factor influencing the efficacy of PACT. Photosensitizers (or their metabolites) can selectively accumulate at sites of infection and undergo a photodynamic reaction when excited by light at specific wavelengths. However, commonly used photosensitizers in antimicrobial applications have several drawbacks, including non-natural origins, unclear metabolic pathways, poor lipid solubility, and high toxicity, which limit their clinical applicability (Guo et al., 2024). The photosensitizer 5-aminolevulinic acid (ALA) is a naturally occurring amino acid precursor to heme and protoporphyrin IX. ALA exhibits enhanced amphiphilicity, allowing it to penetrate cell membranes more efficiently and rapidly, facilitating swift cellular uptake. This results in more precise targeting after treatment. Additionally, ALA displays lower cytotoxicity and favorable photodynamic effects (Fujino et al., 2016).

Therefore, ALA-mediated photodynamic therapy has become an important branch of photodynamic medicine, with a significant emphasis on its basic research (Wang et al., 2020). However, its application in the treatment of osteomyelitis infections remains limited, highlighting the urgent need for further in-depth and detailed research.

Imaging techniques play a crucial role in diagnosing orthopedic diseases, offering unique advantages in diagnosing osteomyelitis. Micro-computed tomography (micro-CT), a non-destructive, three-dimensional imaging technology, enables high-resolution X-ray imaging of living animal models. Using post-processing software, it permits quantitative analysis of bone parameters, facilitating its wide use in bone tissue engineering research. Micro-CT has become the “gold standard” for evaluating bone morphology and microstructure. Imaging-based monitoring can provide insights into changes in bone quality parameters in osteomyelitis from a microscopic perspective (Li et al., 2022), enabling better assessment of treatment efficacy. Therefore, this study used MRSA as an inducer to establish a rabbit tibial osteomyelitis model, and the therapeutic effects of ALA-based PDT alone and in combination with antibiotics were explored using micro-CT, X-ray, bone density measurement, and histopathologic examination, providing new insights and approaches for the anti-infective treatment of osteomyelitis. The findings also offer a foundation for the further expansion of the clinical applications and indications of ALA.

## Materials and methods

### Chemical drugs and lasers

ALA was obtained from Shanghai Macklin Biochemical Co., Ltd. (purity ≥98%, M834743, Shanghai, China). A 5% cod liver oil sodium solution was purchased from Shanghai Xinyi Jinzhu Pharmaceutical Co. (Shanghai, China). Gentamicin, a broad-spectrum antibiotic, was provided by Shiyao Group Ouyi Pharmaceutical Co. (Shijiazhuang, China). A 650-nm semiconductor laser (WSLS-650–500 m-M-2) was procured from Wave Spectrum Laser Group Limited (China).

### Bacterial strain

MRSA was provided by the Laboratory Department of the Affiliated Hospital of Hebei University, and its preparation, antimicrobial spectrum, and bacterial solution concentration followed those described by Yin et al. (2022).

### Fractional inhibitory concentration index (FICI)

Gentamicin and ALA were dissolved in ultrapure water to obtain concentrations of 4×, 2×, 1×, 0.5× and 0.25× relative to the minimum inhibitory concentration (MIC) to determine the FICI. Using a checkerboard design, 50 μL of each drug were added at different concentrations to the horizontal and vertical columns of a 96-well plate. Then, 100 μL of bacterial suspension (1 × 106 CFU/mL) were added and mixed by gentle shaking. These plates were incubated in the dark at 37°C for 30 min followed by 30 min in the light. Next, the samples were incubated in the dark at 37°C for 16–20 h. The MIC of each antimicrobial agent in the combination was determined using the aforementioned method. For each antimicrobial combination, we calculated the FICI by determining the ratio of the MIC of the combination to the MIC of each individual antimicrobial and then summing the two values (see equation:FICI = [MICA (withB)/MICA (alone)] + [MICB (withA)/MICB (alone)]. alone)]). The following criteria were used to interpret the FICI results: synergy, FICI ≤0.5; no interaction, FICI 0.5–4.0; antagonism, FICI >4.0 (Willis et al., 2022).

### Analysis of the relative viability of MRSA suspensions

In total, 6 µL of SYTO9 dye and 6 µL of PI dye were added to 2.0 mL of sterile deionized water and mixed well, and the MRSA suspension was adjusted to 2 × 10^7^ CFU/mL. The live and dead bacteria were treated with saline and 70% isopropanol, respectively, and the suspensions were prepared with five live–cell ratios: 0%, 10%, 50%, 90%, and 100%. Then, 100 µL of the suspension and 100 µL of the staining solution were added to each well. Meanwhile, 100 µL of the suspension and 100 µL of the staining solution were added to 96-well plates, which was incubated at room temperature and protected from light for 15 min, and the fluorescence intensity at 530 (green) and 630 nm (red) was measured using a fluorescence spectrometer at an excitation wavelength of 485 nm. The proportion of live cells in the MRSA suspensions was plotted according as follows: RatioG/R = Fcell, em1/Fcell, em2. The RatioG/R value of the samples was calculated. RatioG/R values to obtain the corresponding percentage of live bacteria.

### Animal experiment

Forty-eight male *New Zealand white rabbits* aged 6 months (average weight: 2.5–3.0 kg) were purchased from Beijing Specific Bio-Technology Co. Ltd. (License No.: SCXK (Beijing) 2019-0010). The rabbits were housed individually in cages under specific pathogen-free conditions. All experimental procedures were conducted in accordance with the National Institutes of Health Guide for the Care and Use of Laboratory Animals, and the protocol was approved by the Laboratory Animal Management Committee/Laboratory Animal Welfare Ethics Committee of Hebei University (Approval No. HBU2024RA021). The room was maintained at an ambient temperature of 22°C ± 2°C and relative humidity of 40%–70%, and the animals were provided with food and sterile water at regular intervals as per the experimental requirements.

### Osteomyelitis models and grouping

The process of osteomyelitis model preparation was reported by Yin et al. (Yin et al., 2022). After surgery, 48 experimental rabbits with bilateral lower limb modeling were randomly divided into four groups (n = 24/group) using the random number table method. One day after modeling, the experimental rabbits in each group began to display signs of immobility, elevated body temperature, depression, decreased feeding, emaciation, and gradual swelling and pus discharge from the wounds. The general condition of the animals and their wounds were continually monitored. On the fifth day of modeling, three animals were randomly selected from each group and anesthetized using 3% pentobarbital solution (30 mg/kg). A surgical incision was created, and a small portion of bone marrow tissue was taken for bacterial culture (the gold standard for identifying osteomyelitis), which was identified as *S. aureus*, indicating that the modeling had been successfully completed.

### Osteomyelitis treatment

Four treatment groups were created: model group (control group) (local injection of 0.2 mL of saline every other day), antibiotics group (intramuscular injection of 16 mg/kg gentamicin sulfate every other day), PACT group (local injection of 0.2 mL of ALA solution [2.0 mM] every other day with laser irradiation performed after 30 min of incubation in the dark once per day), and PACT + antibiotics group (intramuscular injection of 16 mg/kg gentamicin sulfate every other day combined with local injection of 0.2 mL of ALA solution [2.0 mM] every other day and laser irradiation after 30 min of incubation in the dark once per day). In this study, 2.0 mM ALA, the MIC measured in the *in vitro* experiment, was chosen as the therapeutic dose.

Using a semiconductor laser (WSLS-650–500 m-200 M-H4), wounds were exposed to a light source (650 nm) with a power density of 158 mW/cm^2^ for 10 min to achieve a luminous flux of 95 J/cm^2^ (Yin et al., 2022). After illumination, the animals were moved to a dark environment for rearing. To ensure complete absorption of the photosensitizer, the drug was administered every other day for four doses along with daily exposure to light. Animals in each group were treated for 8 days.

#### Detection indicators

##### General condition

The general conditions of the experimental rabbits, such as their mental status, activity, and feeding, were observed and recorded before and every day after the operation. The survival of the animals in each group was monitored and recorded in detail every day after surgery until the end of the experiment.

##### Gross wound and bone anatomy observation

Postoperatively, the experimental rabbits were regularly observed, and the presence or absence of erythema at the incision site, wound exudation, sinus formation, and pus effusion was recorded.

One week after the completion of treatment, the experimental rabbits were euthanized using the air embolization method. The surrounding soft tissues were examined, and the original incision was extended to separate the tissues. The tibia was exposed, and the presence of tibial backbone thickening and severe bone destruction or resorption was assessed. The modified Tang Hui score (Hui et al., 2009) was used for double-blind scoring of the visual observations. The scoring criteria are summarized in Table 1.

**TABLE 1:** Tanghui's score.

0-3 points	0 points 0.5 points 1.0 points 1.5 points 2.0 points 2.5 points 3 points	no signs of infection only a small amount of erythema on the surface of the skin erythema on the surface of the skin and combined with pus oozing erythema on the surface of the skin and combined with sinus formation erythema on the surface of the skin and combined with thickening of the tibial stems mild bone destruction with abscess severe bone destruction with abscess

A score of ≥2 was defined as a significant infection.

##### X-ray observation and evaluation

One week after the completion of treatment, the experimental animals in each group were anesthetized as described for modeling, and the anesthetic dose was adjusted according to the irradiation time. Immediately thereafter, the anesthetized animals were subjected to X-ray scanning to obtain lateral images of the tibia with the following scanning parameters: 50 kV, 100 mA, and 25 ms. These images were used to assess the bone destruction of the tibia and perform radiologic analysis. The severity of osteomyelitis was graded using the modified Norden osteomyelitis scale (Norden et al., 1986), which ranges from 0 to 7, representing the severity of osteomyelitis from absent to present. The details are presented in Table 2.

**TABLE 2:** Norden osteomyelitis score.

	Have	Suspicious	No
Dead bone formation Destruction of bone Hyperosteogeny Soft tissue shadow	3.0 2.0 1.0 1.0	1.5 1.0 0.5 0.5	0 0 0 0

##### Micro-CT evaluation

One week after the completion of treatment, the tibiae of animals in each experimental group were scanned layer-by-layer using the Quantum FX Micro-CT system with the following scanning parameters: scanning voltage, 70 kV; current, 100 μA; exposure time, 3 min; and resolution, 59 μm. In addition, the corresponding tomographic images were obtained. The region of interest (ROI) was set approximately 1 mm below the epiphyseal growth plate, and a length of 1 mm was taken as the ROI. The ROI was outlined using Analyzer15 software to calculate bone mineral density (BMD), trabecular thickness (Tb.Th), and other parameters in each group. The most meaningful bone parameter measurements were selected for comparative analysis with reference to relevant studies (Zhang et al., 2019; Li et al., 2022; Zhao et al., 2024), including: BMD, BoneVolume (BV), TrabeculaeVolume (Tb.BV), Cortical BMD (Cort.BMD), Trabecular BMD (Tb.BMD), Tb.Th, and Trabecular Separation (Tb.Sp).

##### Total body BMD evaluation

One week after the completion of treatment, the experimental animals in each group were anesthetized as described for modeling, and the anesthetic dose was adjusted according to the irradiation time. Immediately thereafter, whole-body bone scans of the anesthetized animals were taken using a dual-energy X-ray bone densitometer. The scanning parameters were as follows: scanning voltage, 76 kV; current, 0.150 mA; exposure time, 4.35 min; and dose, 1.8 μGy.

##### Histologic evaluation

After completing other tests, bone tissue (approximately 1.5 cm in length) was taken from area of bone infection in the tibia of each rabbit, and the collected bone samples were fixed in 4% paraformaldehyde solution at room temperature. After fixation, the samples were subjected to HE staining through a series of steps including dehydration, wax embedding, sectioning, and dewaxing. Stained sections were used to examine inflammatory cells, necrotic tissue, bone destruction, and repair or healing of bone tissue. The initial analysis of osteomyelitis was performed using the modified Smeltzer score method (Smeltzer et al., 1997), the specific scoring rules are detailed in Table 3.

**TABLE 3:** Smeltzer’s histological score.

Score	Acute inflammation	Chronic inflammation	Periosteum inflammation	Osteonecrosis
0	No Inflammation	No Inflammation	No Inflammation	No Osteonecrosis
1	Mild inflammation, No intramedullary abscess	Mild inflammation, no obvious intramedullary fibrosis	Mild inflammation without formation of subperiosteal abscess	One necrotic focus, no dead bone formation
2	Moderate inflammation, no intramedullary abscess	Moderate to severe chronic inflammation, no obvious intramedullary fibrosis	Moderate to severe inflammation without formation of subperiosteal abscess	Multiple necrotic foci, no dead bone formation
3	Mild inflammation with intramedullary abscess	Mild inflammation with intramedullary fibrosis	Mild inflammation with subperiosteal abscess Formation	Single dead bone formation
4	Severe inflammation with intramedullary abscess	Moderate to severe chronic inflammation with intramedullary fibrosis	Moderate to severe inflammation with subperiosteal abscess formation	Multiple dead bones formation

##### Statistical analysis

All recorded data were entered into the computer to organize the database. SPSS 25.0 statistical software was used to analyze the data, and the measurement data were expressed as the mean ± standard deviation. The groups were compared using one-way ANOVA and the least significant difference method. If the variance was not equal, then the Games–Howell test was chosen to compare the groups. Significance was indicated by P < 0.05.

## Results

### Fractional inhibitory concentration index

As presented in Table 4, the mean FICI for the combination treatment was 0.5, denoting a synergistic effect.

**TABLE 4:** Synergistic effects of ALA-PACT with gentamicin.

Bacterial Strain	ALA-PDT		Gentamicin		FIC index	Effect
	MIC_A_	MIC_A (with B)_	MIC_B_	MIC_B (with A)_		
Methicillin-resistant *S. aureus*	2mM	0.5 mM	16 μg/mL	4 μg/mL	0.5	synergy

FIC index of ≤ 0.5 = synergy, >0.5 - < 4 = no interaction and ≥ 4 = antagonism.

Synergy, FICI ≤0.5; no interaction, FICI 0.5–4.0; antagonism, FICI >4.0.

### Analysis of relative viability of MRSA suspensions

**FIGURE 1:**
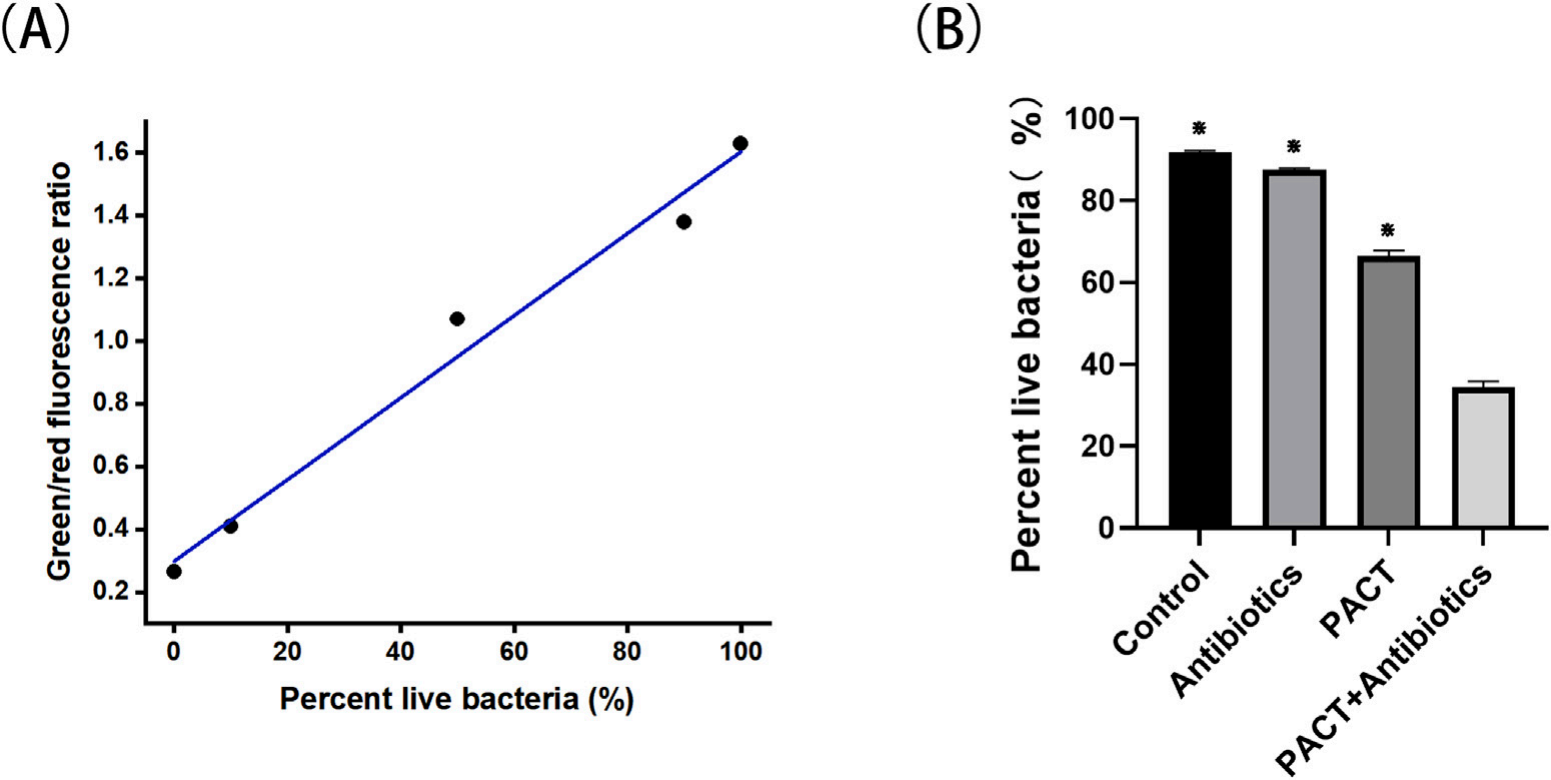
The proportion of live cells in MRSA suspensions and the percent of live bacteria. **(A)** The proportion of live cells in MRSA suspensions. **(B)** The percentage of live bacteria. ⋇P < 0.05 represents a statistically significant difference between the groups (compared with the PACT + antibiotics group).

The proportion of live cells in MRSA suspensions is plotted in Figure 1A, and RatioG/R of the samples was compared with the values in Figure 1A to calculate the corresponding percentage of viable bacteria (Figure 1B). The results revealed a significant decrease (P < 0.05) in cell survival in the combined treatment group compared with the findings in the PACT alone and antibiotics alone groups. This result implies that the combined treatment produced a synergistic effect beyond the sum of the effects of the individual treatments.

### General condition record observation

**FIGURE 2:**
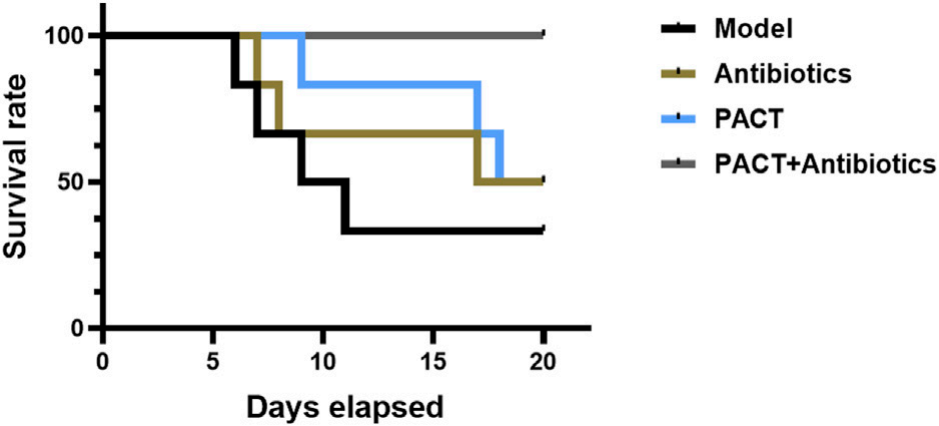
Trends in survival rates by group.

Before modeling, the animals were in good condition, exhibiting normal spirit, activity, and feeding and no obvious differences among the groups. In the first 3 days after modeling, the animals’ feeding and activity were obviously affected, However, there were no significant differences. With prolonged treatment, the animals in each group gradually showed more pronounced differences. The animals in the model and antibiotic groups exhibited a significant reduced mobility, depressed spirit, poor feeding, swelling of the affected limbs, and pus and oozing from the wounds, whereas the animals in the PACT and PACT + antibiotics groups displayed an increase in activity and a gradual increase in food intake, as well as gradual wound healing. The survival curve (Figure 2) illustrated that no animals died in the PACT + antibiotics group Conversely, the survival rates were 50% in the antibiotics and PACT groups and 33% in the model group.

### Gross wound and bone anatomy observation

**FIGURE 3:**
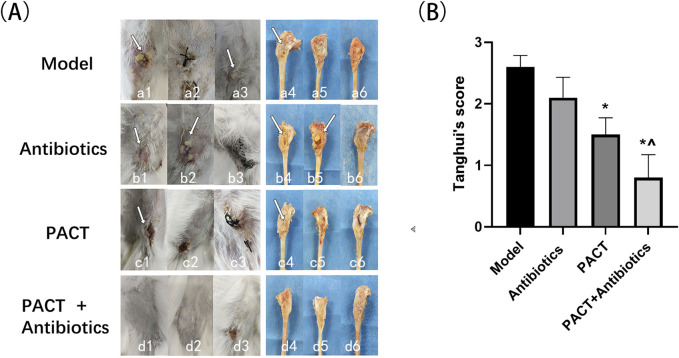
Visual inspection of wound soft tissue, bone tissue, and Tang Hui’s score test results. **(A)** Naked eye observation of soft tissue and bone tissue in each group.; Figure a1: sinus tract formation (white arrow); a3: purulent discharge (white arrow); a4: bone destruction (white arrow); b1: sinus tract formation (white arrow); b2: purulent discharge and exudate (white arrow); b4: bone destruction (white arrow); b5: pus (white arrow); c1: wound crusting (white arrow); c4: bone destruction (white arrow). **(B)** Visual assessment of Tang Hui’s score in each group. *P < 0.05 represents a statistically significant difference compared with the model group). ^ P < 0.05 represents a statistically significant difference compared with the antibiotics group.

Gross observation allows for a visual assessment of the severity of osteomyelitis infection and the effectiveness of treatment. After treatment, the model group exhibited swelling of the soft tissues around the wound, pus discharge, partial sinus tract formation, and obvious bone destruction upon dissection, indicating more severe infection. In the antibiotics group, swelling of the soft tissues, significant pus discharge and oozing, partial sinus tract formation, and bone destruction were observed after dissection, with minimal differences in infection severity compared with that in the model group. In the PACT group, mild swelling around the wound, partial scabbing, and healing were noted. Bone destruction was partially visible upon dissection, but the infection was less severe than those in the model and antibiotics groups. In the PACT + antibiotics group, the soft tissue surface displayed healing after treatment, and there was no obvious bone destruction. The tibia was slightly thickened, and osteomyelitis appeared to be healing (Figure 3A).

Quantitative scoring of soft tissue swelling, abscess formation, sinus tract development, and bone destruction permitted a more systematic statistical analysis. The scores of the treatment groups were all lower than that of the model group, with the score being lowest in the PACT + antibiotics group. Based on the visual scores, the severity of infection decreased in the order of model group > antibiotics group > PACT group > PACT + antibiotics group. The score in the PACT + antibiotics group significantly differed from those in the model and antibiotics groups (P < 0.05), and the score in the PACT group significantly differed from that in the Model group (P < 0.05, Figure 3B).

### X-ray observation and evaluation

**FIGURE 4:**
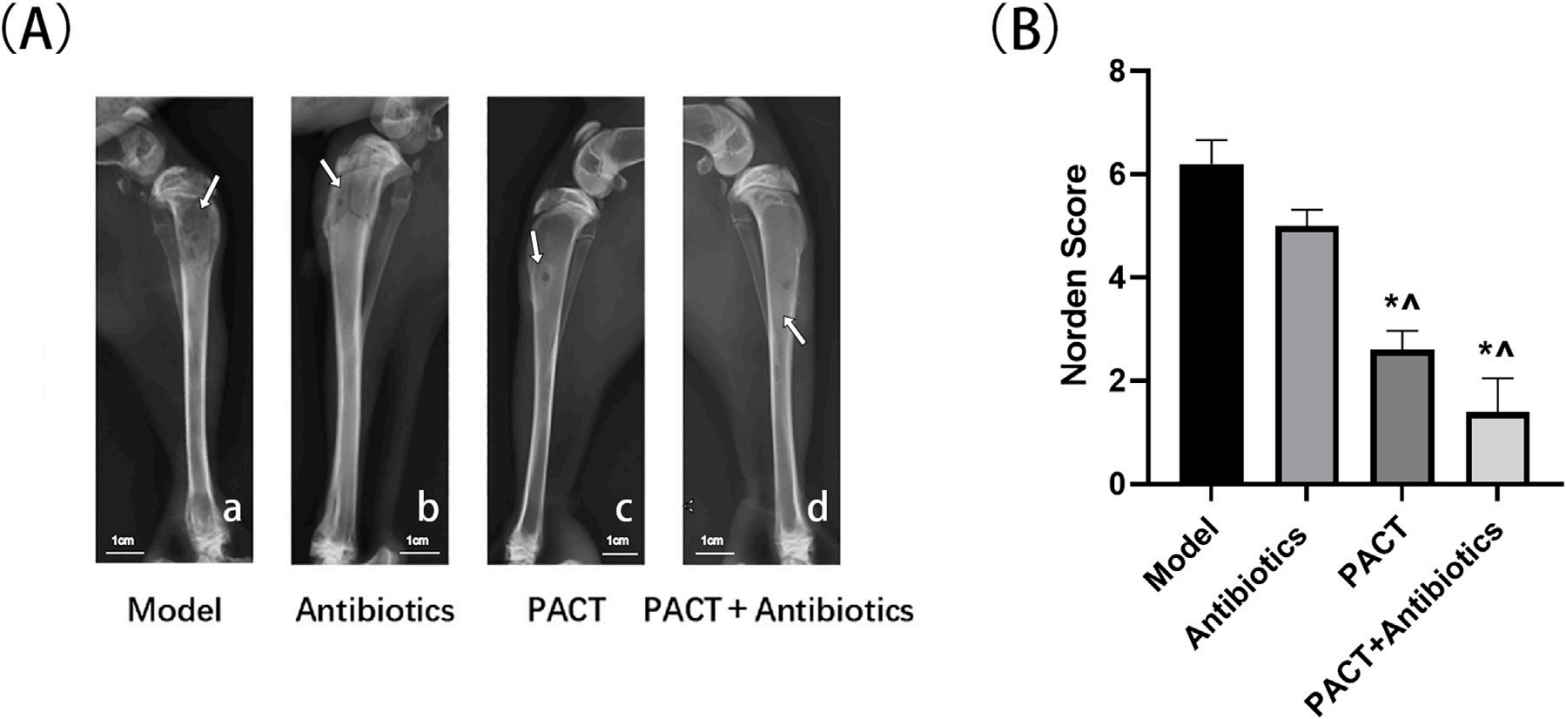
Results of X-ray analysis and the Norden score. **(A)** X-ray assessment in each group.; (A-a): Area of low density bone destruction (white arrow); (A-b): Pathologic fracture (white arrow); (A-c): Circumferential sclerotic rim around bone defect (white arrow); (A-d): Sclerotic osteophytes with mild thickening of the diaphysis (white arrow) **(B)** Norden score in each group. *P < 0.05 represents a statistically significant difference compared with the model group. ^P < 0.05 represents a statistically significant difference compared with the antibiotics group.

At the end of treatment, the X-ray results illustrated that the original bone defect did not heal in the model group, with obvious bone destruction, hyperplasia and sclerosis of bone, partial osteonecrosis, and slight swelling of the surrounding soft tissues. In the antibiotics group, the area of the original bone defect was slightly indistinct together with hyperplasia and sclerosis of bone and a little laminar periosteal reaction, with some areas displaying pathologic fractures. In addition, the surrounding soft tissues were obviously swollen with a small amount of air accumulation. In the PACT group, there was a ring-shaped sclerotic border around the bone defect, slight thickening of the tibial backbone, obvious periosteal reaction, and slight swelling of the soft tissues. In the PACT + antibiotics group, there was no obvious bone destruction, the bone defect was indistinct, and there was sclerotic osteoid hyperplasia around the bone defect. Other findings included slight thickening of the tibial backbone, a periosteal reaction around the cortical bone, and swelling in the surrounding soft tissues (Figure 4A).

Bone destruction, osteophytes, osteonecrosis, and soft tissue swelling were scored semi-quantitatively, and the modified Norden scores illustrated that the severity of bone destruction decreased in the order of model group > antibiotics group > PACT group > PACT + antibiotics group. The severity was significantly lower in the PACT + antibiotics and PACT groups than in the model and antibiotics groups (P < 0.05, Figure 4B).

### Micro-CT observation

Micro-CT has been widely used in the study of animal bone tissue because the scanned tomographic image can be both observed and evaluated. Moreover, the bulk image information obtained by micro-CT can be used to select the ROI and perform threshold segmentation using analysis software, followed by post-processing to analyze the data on the microstructure of the bone to better evaluate the changes of the bone.

**FIGURE 5:**
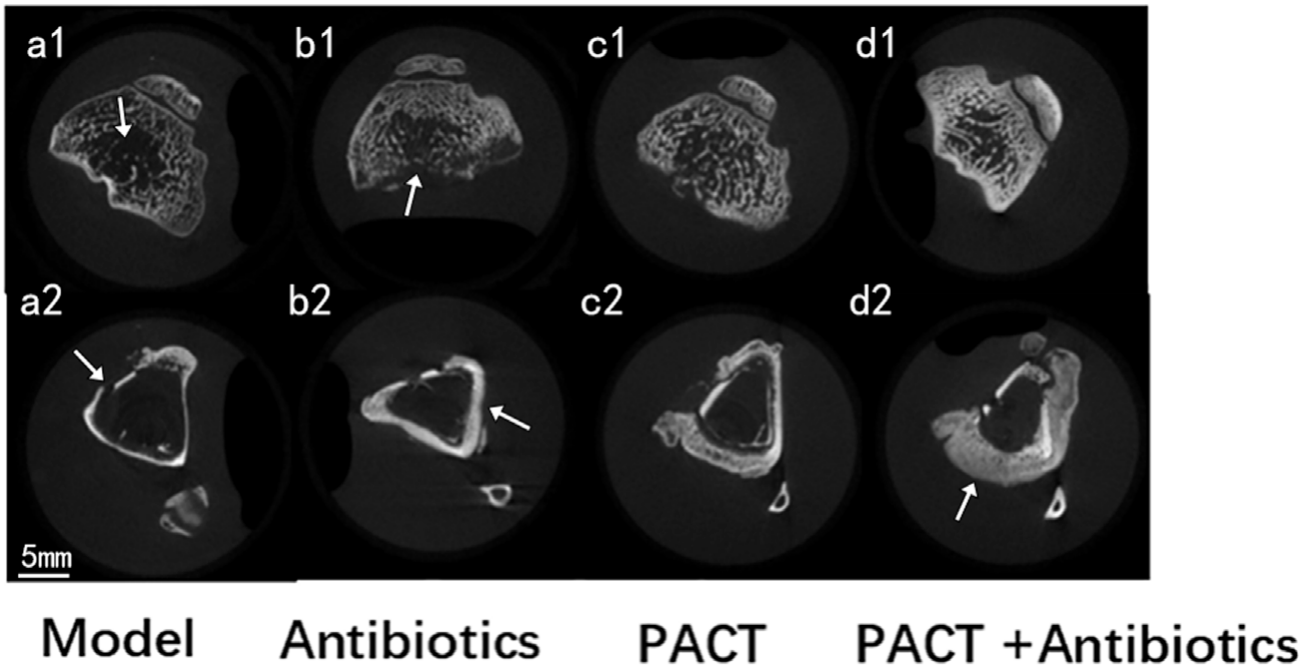
Micro-CT images of each group.; a1: thinning and destruction of cancellous bone; b1: thinning and defective bone cortex; a2: destruction and defective bone cortex; b2: thickening of the backbone; d2: marked periosteal reaction.

Micro-CT scans of the condylar and backbone regions are presented in Figure 5, and the overall observation of the condylar region illustrated that the cancellous bone structure of each group displayed varying degrees of sparse and destruction, thinning of the bone cortex, as well as defects. In the PACT + antibiotics group, the anatomical structure of the image was clear, the contrast and sharpness were good, the cancellous bone was more homogeneous, and the cortical bone was intact. Observation of the structure at the level of the backbone in the modeling area illustrated that the treatment groups had obvious bone hyperplasia, different degrees of thickening of the backbone, and more obvious periosteal reaction, especially in the PACT and PACT + antibiotics groups.

### Micro-CT bone parameter analysis

**FIGURE 6:**
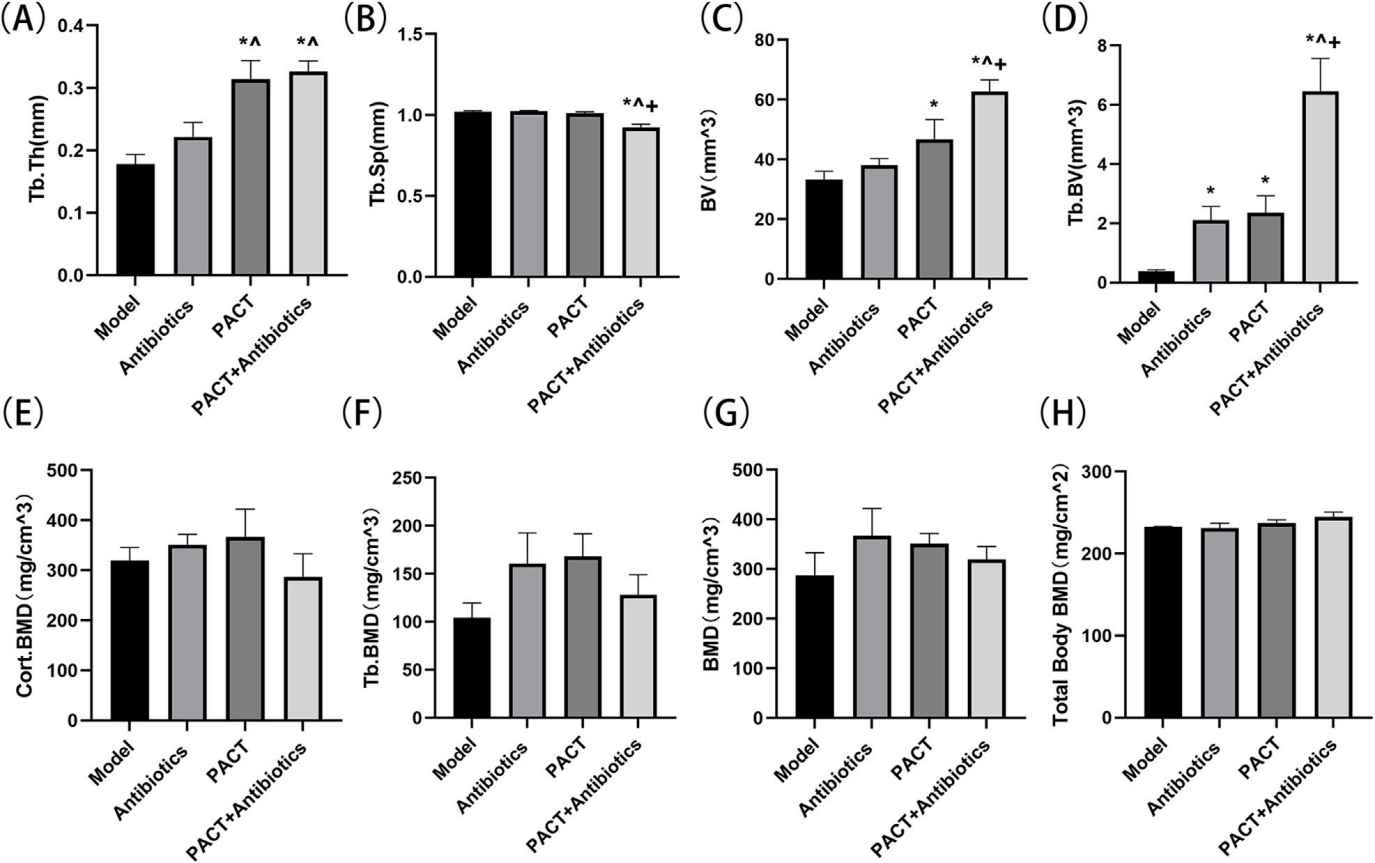
Micro-CT detection of the effects of different treatment modalities on bone structural parameters. **(A)** Tb.Th **(B)** Tb.Sp. **(C)** BV **(D)** Tb.BV. **(E)** Cort.BMD **(F)** Tb.BMD. **(G)** BMD **(H)** Total Body BMD. *P < 0.05 represents significant differences compared with the model group. ^P < 0.05 indicates significant differences compared with the antibiotics group. +P < 0.05 indicates significant differences compared with the PACT group.

On micro-CT, Tb.Th was significantly higher in the PACT and PACT + antibiotics groups than in the model and antibiotics groups (P < 0.05), and the value was lower in the model group than in the antibiotics group (P > 0.05, Figure 6A). Tb.Sp was significantly lower in the PACT + antibiotics group than in the other groups (P < 0.05, Figure 6B). The total bone volume and trabecular bone volume were also significantly higher in the PACT + antibiotics group than in the other groups (P < 0.05). The PACT group displayed significant differences compared with the model group (P < 0.05, Figures 6C, D). Additionally, the trabecular bone volume was significantly higher in the antibiotics group than in the model group (P < 0.05). No statistically significant differences were found in overall BMD, cortical BMD, and trabecular BMD among the groups (P > 0.05, Figures 6E–G).

### Total body BMD evaluation

The Total body BMD ranged 0.23–0.24 g/cm^2^ in each group. The value was higher in the PACT + antibiotics group than in the PACT group, whereas the value was similar between the model and antibiotics groups. No significant statistical differences were observed between the groups (P > 0.05, Figure 6H).

### Histologic evaluation

**FIGURE 7:**
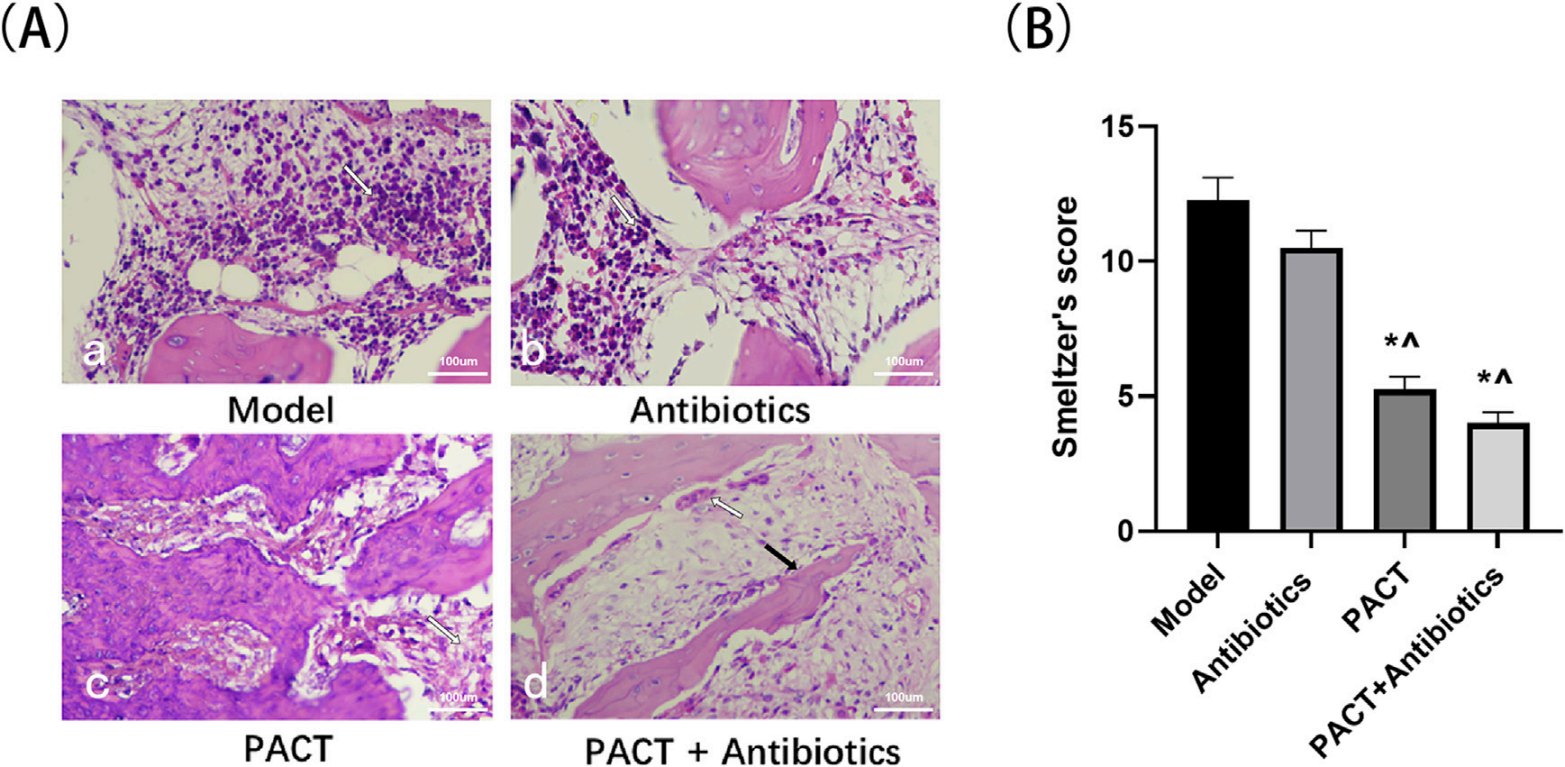
Pathologic results and Smeltzer pathology scores of each group. **(A)** HE staining results of bone tissues (×20).; (A-a): Small abscess (white arrow); (A-b): Inflammatory cells (white arrow); (A-c): Fibrosis (white arrow); (A-d): Periosteal reaction (white arrow), newborn bone (black arrow). **(B)** Smeltzer’s scores of bone tissues. *P < 0.05 denotes significant differences compared with the model group). ^ P < 0.05 denotes significant differences compared with the antibiotics group.

Histology was performed to evaluate the therapeutic effect of PACT on osteomyelitis at a microscopic level. One week after the completion of treatment, the model group displayed obvious bone inflammation, including obvious inflammatory cell infiltration in bone tissue. In addition, multiple small abscesses had formed, and part of the bone was destroyed. The antibiotics group extensive many inflammatory cell infiltration, the formation of multiple small abscesses, and fibrous tissues in the bone marrow cavity. The PACT group had a low level of inflammatory cell infiltration, fibrous tissues and small necrotic foci were present in the bone marrow cavity, and some neoplastic bone had formed. The PACT + antibiotics group exhibited a small amount of inflammatory cell infiltration in bone tissue, and fibrous tissue was observed in the bone marrow cavity. In addition, a large number of strips and pieces of new bone formation were present in the field of view, and periosteal hyperplasia was more obvious (Figure 7A).

The histologic scores were significantly lower in the PACT + antibiotics and PACT groups than in the model and antibiotics groups (P < 0.05, Figure 7B).

## Discussion

Osteomyelitis remains a persistent challenge in medicine. This condition is often associated with circulatory disorders, immune suppression, and bacterial resistance (Masters et al., 2022). Traditional antibiotic therapies are significantly less effective because of the persistence of inflammatory responses, whereas the success rate of surgical treatments is unsatisfactory because of recurrent infections and impaired bone tissue regeneration (Ranjbar and Alam, 2023). This is particularly true for patients with diabetic, traumatic, or post-surgical osteomyelitis, in whom recurrent infections complicate treatment strategies. Advanced osteomyelitis often leads to severe bone destruction and dysfunction, exacerbating the disease and increasing the consumption of medical resources (Colston and Atkins, 2018).Given these factors, identifying an effective treatment or method to improve osteomyelitis management is of great interest and importance.

Suitable animal models are crucial for the success of medical research (Huang et al., 2023). In this experiment, *New Zealand white rabbits* were used as the experimental animals, and a small hole was made in the tibia, followed by the injection of bacteria and a vascular sclerosing agent to induce osteomyelitis (Norden, 1970). MRSA is emerging as an important causative agent of nosocomial and community-acquired infections because of the use of antibiotics in high doses and frequencies (Lee et al., 2018; Turner et al., 2019). Studies indicated that antibiotic resistance could lead to millions of deaths by 2050 (Sutradhar et al., 2021), underscoring the urgent need to address infections caused by drug-resistant bacteria.

**SCHEME 1:**
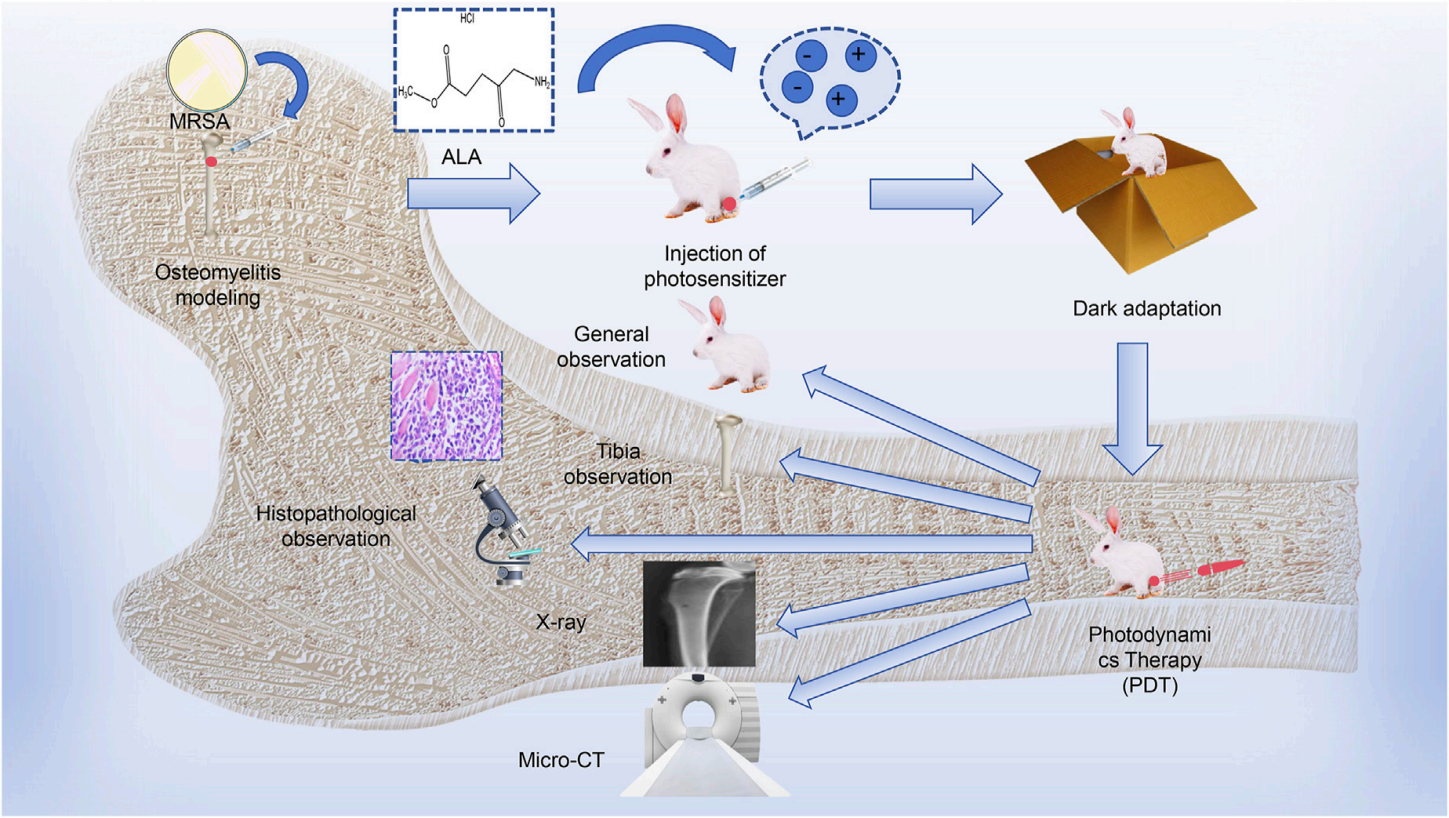
The process of PACT in rabbits with tibial osteomyelitis.

The process of this study is described in Scheme 1. The results of this study revealed no significant differences among the groups. After treatment, the animals in the model and antibiotic groups displayed obvious reduced mobility and reduced feeding, whereas the overall activity and mental state of animals was better in the PACT and PACT + antibiotics groups. At the same time, the degree of infection was lower in the PACT group than in the model and antibiotics groups after treatment, whereas the soft tissue surface healed well in the PACT + antibiotics group after the completion of treatment, as well as no obvious bone destruction, slight thickening of the bone stem, and healing of osteomyelitis lesions. This suggested that PACT represents a new anti-infective strategy relative to traditional antibiotic treatment. Antibiotic treatment displayed a certain sensitizing effect when combined with PACT because this combination effectively halted the progression of osteomyelitis. This effect might be explained by the fact that PACT promotes local blood circulation, which in turn increases the penetration of antibiotics into the wound (Zhao et al., 2024). The percentage of viable bacteria showed that the percentage of cell membrane-damaged cells in the PACT + antibiotic group was significantly higher than the sum of the percentages in the PACT and antibiotic groups, suggesting that PACT disrupted the bacterial cell membranes, making it easier for gentamicin to enter into the interior of the MRSA, and further destroying the integrity of the cell membranes. This may be one of the mechanisms by which PACT and gentamicin act synergistically. Meanwhile, the results of survival analysis indirectly proved this experimental hypothesis.

Imaging has always played an important role in the evaluation of osteoarthritic diseases (Park and Fritz, 2023). X-ray can provide a general understanding of the severity of bone infection because it can highlight bone destruction and resorption, thickening of the tibial tuberosity, and swelling of the surrounding soft tissues. X-ray performed after treatment revealed blurring of bone defects, better bone healing, and slight swelling of the surrounding soft tissues in the PACT + antibiotics group, and these outcomes were better than those in the PACT and antibiotics groups. By contrast, the untreated model group featured obvious bone destruction, proliferation and hardening of bone, osteonecrosis, and swelling of the surrounding soft tissues. Meanwhile, the Norden score indicated that the disease severity decreased in the order of model group > antibiotics group > PACT group > PACT + antibiotics group (P < 0.05). The same trend was noted for the Tang Hui score and pathohistologic score in the bulk observation.

Micro-CT can be used to observe the microstructure of bone joints in detail and effectively assess the three-dimensional structure of bone tissue (Piscaer et al., 2008; Tan et al., 2022).It can additionally be used to assess the bone healing process, contributing to a deeper understanding of the mechanism of action and therapeutic effects of drugs (Wee et al., 2022). Imaging of the anatomical structure demonstrated that the PACT + antibiotics group had more uniform cancellous bone, more complete bone cortex, and the best continuity of the structural morphology of cancellous and cortical bone. Further analysis using post-processing software permitted visual and quantitative analyses of the structure and quality of bone after measuring several bone parameters (Suzuki et al., 2018; Li et al., 2022). Tb.Th and Tb.Sp are micro-CT bone parameters used to evaluate the internal structural characteristics of cancellous bone from a microscopic perspective because these variables sensitively reflect the morphology and structural alterations of the trabeculae of cancellous bone caused by inflammation. Tb.Th and Tb.Sp did not differ between the model and antibiotics groups, but a difference was detected between the PACT and PACT + antibiotics groups (P < 0.05), indicating that the PACT treatment effectively improved the thickness of trabecular bone. In addition, Tb.Th was highest and Tb.Sp was lowest in the PACT + antibiotics group, consistent with the anatomical findings on micro-CT, indicating that PACT + antibiotics could stop the progression of osteomyelitis by improving the internal structure of bone.

Bone volume is an important variable of bone morphology that reflects the development of bone, and it is also closely related to the mechanical properties of bone. The total bone volume and trabecular bone volume were higher in the PACT + antibiotics group than in the other groups (P < 0.05), and they were higher in the PACT group than in the model group (P < 0.05). The trabecular bone volume was higher in the antibiotics group than in the model group (P < 0.05). These findings indicated that the treatment effectively stimulated the proliferation of bone tissue, causing changes in volume. In particular, the efficacy of PACT and PACT + antibiotics was significant, suggesting that these two treatment regimens have positive effects on bone repair and regeneration. However, no significant differences were observed among the groups regarding the local tibia overall BMD, cortical BMD, and trabecular BMD as measured by micro-CT and whole-body BMD measured by bone densitometry (P > 0.05). Thus, it cannot be stated that PACT altered bone density in this experiment.

The periosteum surrounds most skeletal structures. The osteogenic potential of the periosteum is activated in several pathologic conditions, such as infections, benign and malignant tumors, and systemic diseases, resulting in the production of new bone tissue, a process also termed the periosteal reaction or periosteal neo-osteogenesis (Bisseret et al., 2015). Periosteal reaction is a natural phenomenon in the process of bone healing that is characterized on imaging by periosteal thickening or the presence of new bone formation. According to related studies, periosteal reaction is closely related to the healing and repair processes of bone tissue (Lin et al., 2014; Allen et al., 2023).

Micro-CT revealed different degrees of periosteal reaction were observed in all experimental groups (Figure 5). Compared with the findings in the model group, periosteal hyperplasia were more obvious in the PACT and PACT + antibiotics groups, and PACT enhanced the osteogenic activity of the periosteum, increased the anabolism of the osteocortex, and activated bone healing, especially in the PACT + antibiotics group. The pathohistologic results indicated that inflammatory cell infiltration was greater in the model and antibiotics groups. There was obvious intraosseous inflammation in the model group, whereas there were fewer inflammatory cells in the PACT and PACT + antibiotics groups, as well as some strips of flaky new bone. In addition, periosteal hyperplasia was more obvious in the PACT + antibiotics group.

Based on the study results, PACT can reduce inflammatory responses in osteomyelitis and accelerate bone tissue repair and regeneration to some extent. PACT displayed efficacy in treating tibial osteomyelitis in rabbits, and its therapeutic effect was significantly enhanced in combination with antibiotics, demonstrating its superior therapeutic potential.

### Shortcomings and prospects

First, the animal model established in this experiment is relatively simple and ideal. However, because of the increased frequency of high-violence injuries such as car accidents in modern society and the aging population, osteomyelitis is more frequently caused by complex trauma, and a significant proportion of events also involve internal fixation devices. The incidence of internal implant-related osteomyelitis, which is characterized by a tendency to form biofilm attachment sites and cause biofilm-related osteomyelitis, is rising, further hindering the efficacy of anti-infective therapy, and the fabrication of an implant-related model should be considered to further validate the anti-microbial efficacy of PACT.

Second, X-ray and micro-CT were used to detect the effects of the treatments against osteomyelitis in this study, but they provide less information than MRI, which permits a more sensitive observation of soft tissues and bone marrow. Thus, MRI should be considered to more comprehensively assess the experimental efficacy of the treatments from the imaging point of view.

Third, we focused on the efficacy of PACT in the treatment of osteomyelitis, but we did not include a separate laser treatment group to explore the effect of low-dose laser exposure on bone healing. This could be assessed by including light-only groups and other photosensitizer-mediated PACT groups for comparative exploratory studies.

## Conclusion

The present study confirmed the therapeutic advantages of ALA-based PACT in the treatment of osteomyelitis caused by drug-resistant bacteria. ALA-based PACT effectively reduced the inflammatory response and ameliorated the destruction of bone microstructure caused by inflammation, thereby promoting the healing of bone tissue. In addition, our findings suggest that the combination of PACT and antibiotics produces specific synergistic effects in the treatment of osteomyelitis, thereby providing a promising new approach for future clinical studies of osteomyelitis treatment.

## Data Availability

The original contributions presented in the study are included in the article/supplementary material, further inquiries can be directed to the corresponding authors.

## References

[B1] Allen H. Barnthouse N. C. Chan B. Y. (2023). Periosteal pathologic conditions: imaging findings and pathophysiology. Radiographics 43 (2), e220120. 10.1148/rg.220120 36525365

[B2] Belthur M. V. Birchansky S. B. Verdugo A. A. Mason E. O. Jr. Hulten K. G. Kaplan S. L. (2012). Pathologic fractures in children with acute *Staphylococcus aureus* osteomyelitis. J. Bone Jt. Surg. Am. 94 (1), 34–42. 10.2106/jbjs.J.01915 22218380

[B3] Bisseret D. Kaci R. Lafage-Proust M. H. Alison M. Parlier-Cuau C. Laredo J. D. (2015). Periosteum: characteristic imaging findings with emphasis on radiologic-pathologic comparisons. Skelet. Radiol. 44 (3), 321–338. 10.1007/s00256-014-1976-5 25269751

[B4] Cierny G. Mader J. T. Penninck J. J. (2003). The classic: a clinical staging system for adult osteomyelitis. Clin. Orthop. Relat. Res. 414, 7–24. 10.1097/01.blo.0000088564.81746.62 12966271

[B5] Colston J. Atkins B. (2018). Bone and joint infection. Clin. Med. (Lond) 18 (2), 150–154. 10.7861/clinmedicine.18-2-150 29626020 PMC6303448

[B6] Cortés-Penfield N. W. Kulkarni P. A. (2019). The history of antibiotic treatment of osteomyelitis. Open Forum Infect. Dis. 6 (5), ofz181. 10.1093/ofid/ofz181 31123692 PMC6524831

[B7] Fujino M. Nishio Y. Ito H. Tanaka T. Li X. K. (2016). 5-Aminolevulinic acid regulates the inflammatory response and alloimmune reaction. Int. Immunopharmacol. 37, 71–78. 10.1016/j.intimp.2015.11.034 26643355

[B8] Guo N. Chen J. Kong F. Gao Y. Bian J. Liu T. (2024). 5-aminolevulinic acid photodynamic therapy for chronic wound infection in rats with diabetes. Biomed. Pharmacother. 178, 117132. 10.1016/j.biopha.2024.117132 39047418

[B9] Hawkshead J. J. Patel N. B. Steele R. W. Heinrich S. D. (2009). Comparative severity of pediatric osteomyelitis attributable to methicillin-resistant versus methicillin-sensitive *Staphylococcus aureus* . J. Pediatr. Orthop. 29 (1), 85–90. 10.1097/BPO.0b013e3181901c3a 19098653

[B10] Huang S. Wen J. Zhang Y. Bai X. Cui Z. K. (2023). Choosing the right animal model for osteomyelitis research: considerations and challenges. J. Orthop. Transl. 43, 47–65. 10.1016/j.jot.2023.10.001 PMC10716383 38094261

[B11] Hui T. Yongqing X. TianEr Z. (2009). Relationship between inoculation dose and rabbit model of staphlococcus osteomyelitis. Orthop. J. China 17, 700–702.

[B12] Jones H. W. Harrison J. W. Bates J. Evans G. A. Lubega N. (2009). Radiologic classification of chronic hematogenous osteomyelitis in children. J. Pediatr. Orthop. 29 (7), 822–827. 10.1097/BPO.0b013e3181b76933 20104169

[B13] Kavanagh N. Ryan E. J. Widaa A. Sexton G. Fennell J. O'Rourke S. (2018). Staphylococcal osteomyelitis: disease progression, treatment challenges, and future directions. Clin. Microbiol. Rev. 31 (2), e00084. 10.1128/cmr.00084-17 PMC5967688 29444953

[B14] Kolarikova M. Hosikova B. Dilenko H. Barton-Tomankova K. Valkova L. Bajgar R. (2023). Photodynamic therapy: innovative approaches for antibacterial and anticancer treatments. Med. Res. Rev. 43 (4), 717–774. 10.1002/med.21935 36757198

[B15] Lari A. Esmaeil A. Marples M. Watts A. Pincher B. Sharma H. (2024). Single versus two-stage management of long-bone chronic osteomyelitis in adults: a systematic review and meta-analysis. J. Orthop. Surg. Res. 19 (1), 351. 10.1186/s13018-024-04832-7 38877562 PMC11177413

[B16] Lee A. S. de Lencastre H. Garau J. Kluytmans J. Malhotra-Kumar S. Peschel A. (2018). Methicillin-resistant *Staphylococcus aureus* . Nat. Rev. Dis. Prim. 4, 18033. 10.1038/nrdp.2018.33 29849094

[B17] Lew D. P. Waldvogel F. A. (2004). Osteomyelitis. Lancet 364 (9431), 369–379. 10.1016/s0140-6736(04)16727-5 15276398

[B18] Li Y. Chen L. Lin M. Wang C. Zhang R. Li Y. (2022). Micro-CT analysis of osteomyelitis of rabbit tibial for model establishment and biomaterials application in tissue engineering. Heliyon 8 (12), e12471. 10.1016/j.heliyon.2022.e12471 36643303 PMC9834739

[B19] Lin Z. Fateh A. Salem D. M. Intini G. (2014). Periosteum: biology and applications in craniofacial bone regeneration. J. Dent. Res. 93 (2), 109–116. 10.1177/0022034513506445 24088412 PMC3895334

[B20] Maldonado-Carmona N. Marchand G. Villandier N. Ouk T. S. Pereira M. M. Calvete M. J. F. (2020). Porphyrin-Loaded lignin nanoparticles against bacteria: a photodynamic antimicrobial chemotherapy application. Front. Microbiol. 11, 606185. 10.3389/fmicb.2020.606185 33281805 PMC7705181

[B21] Masters E. A. Ricciardi B. F. Bentley K. L. M. Moriarty T. F. Schwarz E. M. Muthukrishnan G. (2022). Skeletal infections: microbial pathogenesis, immunity and clinical management. Nat. Rev. Microbiol. 20 (7), 385–400. 10.1038/s41579-022-00686-0 35169289 PMC8852989

[B22] Masters E. A. Trombetta R. P. de Mesy Bentley K. L. Boyce B. F. Gill A. L. Gill S. R. (2019). Evolving concepts in bone infection: redefining “biofilm”, “acute vs. chronic osteomyelitis”, “the immune proteome” and “local antibiotic therapy”. Bone Res. 7, 20. 10.1038/s41413-019-0061-z 31646012 PMC6804538

[B23] Muthukrishnan G. Masters E. A. Daiss J. L. Schwarz E. M. (2019). Mechanisms of immune evasion and bone tissue colonization that make *Staphylococcus aureus* the primary pathogen in osteomyelitis. Curr. Osteoporos. Rep. 17 (6), 395–404. 10.1007/s11914-019-00548-4 31721069 PMC7344867

[B24] Norden C. W. (1970). Experimental osteomyelitis. I. A description of the model. J. Infect. Dis. 122 (5), 410–418. 10.1093/infdis/122.5.410 5476391

[B25] Norden C. W. Shinners E. Niederriter K. (1986). Clindamycin treatment of experimental chronic osteomyelitis due to *Staphylococcus aureus* . J. Infect. Dis. 153 (5), 956–959. 10.1093/infdis/153.5.956 3701108

[B26] Obremskey W. T. Metsemakers W. J. Schlatterer D. R. Tetsworth K. Egol K. Kates S. (2020). Musculoskeletal infection in orthopaedic trauma: assessment of the 2018 international consensus meeting on musculoskeletal infection. J. Bone Jt. Surg. Am. 102 (10), e44. 10.2106/jbjs.19.01070 32118653

[B27] Park E. H. Fritz J. (2023). The role of imaging in osteoarthritis. Best. Pract. Res. Clin. Rheumatol. 37 (2), 101866. 10.1016/j.berh.2023.101866 37659890

[B28] Piscaer T. M. Waarsing J. H. Kops N. Pavljasevic P. Verhaar J. A. van Osch G. J. (2008). *In vivo* imaging of cartilage degeneration using microCT-arthrography. Osteoarthr. Cartil. 16 (9), 1011–1017. 10.1016/j.joca.2008.01.012 18342549

[B29] Qian L. Fei Q. Zhang H. Qiu M. Zhang B. Wang Q. (2020). lncRNA HOTAIR promotes DNA repair and radioresistance of breast cancer via EZH2. DNA Cell Biol. 39, 2166–2173. 10.1089/dna.2020.5771 33136445

[B30] Ranjbar R. Alam M. (2023). Antimicrobial Resistance Collaborators (2022). Global burden of bacterial antimicrobial resistance in 2019: a systematic analysis. Evid. Based Nurs. 27, 16. 10.1136/ebnurs-2022-103540 37500506

[B31] Salik M. Mir M. H. Philip D. Verma S. (2021). Brodie's abscess: a diagnostic conundrum. Cureus 13 (7), e16426. 10.7759/cureus.16426 34422465 PMC8369975

[B32] Smeltzer M. S. Thomas J. R. Hickmon S. G. Skinner R. A. Nelson C. L. Griffith D. (1997). Characterization of a rabbit model of staphylococcal osteomyelitis. J. Orthop. Res. 15 (3), 414–421. 10.1002/jor.1100150314 9246088

[B33] Sobotta L. Skupin-Mrugalska P. Piskorz J. Mielcarek J. (2019). Porphyrinoid photosensitizers mediated photodynamic inactivation against bacteria. Eur. J. Med. Chem. 175, 72–106. 10.1016/j.ejmech.2019.04.057 31096157

[B34] St Denis T. G. Dai T. Izikson L. Astrakas C. Anderson R. R. Hamblin M. R. (2011). All you need is light: antimicrobial photoinactivation as an evolving and emerging discovery strategy against infectious disease. Virulence 2 (6), 509–520. 10.4161/viru.2.6.17889 21971183 PMC3260545

[B35] St Jeor J. D. Thomas K. B. Thacker P. G. Hull N. C. (2020). Multifocal subacute osteomyelitis in adjacent bones in the ankle without septic joint. Radiol. Case Rep. 15 (10), 1927–1930. 10.1016/j.radcr.2020.07.073 32874386 PMC7452080

[B36] Sutradhar I. Ching C. Desai D. Suprenant M. Briars E. Heins Z. (2021). Computational model to quantify the growth of antibiotic-resistant bacteria in wastewater. mSystems 6 (3), e0036021. 10.1128/mSystems.00360-21 34100640 PMC8579810

[B37] Suzuki S. S. Garcez A. S. Reese P. O. Suzuki H. Ribeiro M. S. Moon W. (2018). Effects of corticopuncture (CP) and low-level laser therapy (LLLT) on the rate of tooth movement and root resorption in rats using micro-CT evaluation. Lasers Med. Sci. 33 (4), 811–821. 10.1007/s10103-017-2421-5 29282560

[B38] Tan J. Labrinidis A. Williams R. Mian M. Anderson P. J. Ranjitkar S. (2022). Micro-CT-based bone microarchitecture analysis of the murine skull. Methods Mol. Biol. 2403, 129–145. 10.1007/978-1-0716-1847-9_10 34913121

[B39] Turner N. A. Sharma-Kuinkel B. K. Maskarinec S. A. Eichenberger E. M. Shah P. P. Carugati M. (2019). Methicillin-resistant *Staphylococcus aureus*: an overview of basic and clinical research. Nat. Rev. Microbiol. 17 (4), 203–218. 10.1038/s41579-018-0147-4 30737488 PMC6939889

[B40] Urish K. L. Cassat J. E. (2020). *Staphylococcus aureus* osteomyelitis: bone, bugs, and surgery. Infect. Immun. 88 (7), e00932. 10.1128/iai.00932-19 32094258 PMC7309607

[B41] Ventola C. L. (2015). The antibiotic resistance crisis: part 1: causes and threats. P t 40 (4), 277–283. 25859123 PMC4378521

[B42] Waldvogel F. A. Medoff G. Swartz M. N. (1970). Osteomyelitis: a review of clinical features, therapeutic considerations and unusual aspects. N. Engl. J. Med. 282 (4), 198–206. 10.1056/nejm197001222820406 4902833

[B43] Walter N. Rupp M. Baertl S. Hinterberger T. Alt V. (2022). Prevalence of psychological comorbidities in bone infection. J. Psychosom. Res. 157, 110806. 10.1016/j.jpsychores.2022.110806 35367917

[B44] Wang X. Tian Y. Liao X. Tang Y. Ni Q. Sun J. (2020). Enhancing selective photosensitizer accumulation and oxygen supply for high-efficacy photodynamic therapy toward glioma by 5-aminolevulinic acid loaded nanoplatform. J. Colloid Interface Sci. 565, 483–493. 10.1016/j.jcis.2020.01.020 31982715

[B45] Wee H. Khajuria D. K. Kamal F. Lewis G. S. Elbarbary R. A. (2022). Assessment of bone fracture healing using micro-computed tomography. J. Vis. Exp. 190. 10.3791/64262 PMC10309065 36571411

[B46] Willis J. A. Cheburkanov V. Chen S. Soares J. M. Kassab G. Blanco K. C. (2022). Breaking down antibiotic resistance in methicillin-resistant *Staphylococcus aureus*: combining antimicrobial photodynamic and antibiotic treatments. Proc. Natl. Acad. Sci. U. S. A. 119 (36), e2208378119. 10.1073/pnas.2208378119 36037346 PMC9457041

[B47] Yi J. Wood J. B. Creech C. B. Williams D. Jimenez-Truque N. Yildirim I. (2021). Clinical epidemiology and outcomes of pediatric musculoskeletal infections. J. Pediatr. 234, 236–244.e2. 10.1016/j.jpeds.2021.03.028 33771580 PMC8238832

[B48] Yin X. Fang Z. Fang Y. Zhu L. Pang J. Liu T. (2022). Antimicrobial photodynamic therapy involving a novel photosensitizer combined with an antibiotic in the treatment of rabbit tibial osteomyelitis caused by drug-resistant bacteria. Front. Microbiol. 13, 876166. 10.3389/fmicb.2022.876166 35531297 PMC9073078

[B49] Zairi M. Boussetta R. Msakni A. Mohseni A. A. Nessib M. N. (2021). Subacute osteomyelitis of the tibial diaphysis associated with Brodie's abscess: a rare case report of a four-year-old child. Int. J. Surg. Case Rep. 89, 106453. 10.1016/j.ijscr.2021.106453 34775323 PMC8593451

[B50] Zhang Y. Shen L. Wang P. Xi W. Yu Z. Huang X. (2019). Treatment with vancomycin loaded calcium sulphate and autogenous bone in an improved rabbit model of bone infection. J. Vis. Exp. 145. 10.3791/57294 30933056

[B51] Zhao Z. Pang J. Zhao D. Guo N. Guo Y. Kong F. (2024). Exploring the efficacy of photodynamic antimicrobial chemotherapy on diabetic foot ulcers in rats. J. Biophot. 17 (7), e202300568. 10.1002/jbio.202300568 38651324

